# Molecular fingerprinting by multi-locus sequence typing identifies microevolution and nosocomial transmission of *Candida glabrata* in Kuwait

**DOI:** 10.3389/fpubh.2023.1242622

**Published:** 2023-09-08

**Authors:** Mohammad Asadzadeh, Suhail Ahmad, Noura Al-Sweih, Ziauddin Khan

**Affiliations:** Department of Microbiology, College of Medicine, Kuwait University, Jabriya, Kuwait

**Keywords:** *Candida glabrata*, MLST, microevolution, nosocomial transmission, fingerprinting

## Abstract

**Backgrounds:**

*Candida glabrata* is a frequently isolated non-*albicans Candida* species and invasive *C. glabrata* infections in older patients are associated with high mortality rates. Opportunistic *Candida* infections in critically ill patients may be either endogenous or nosocomial in origin and this distinction is critical for effective intervention strategies. This study performed multi-locus sequence typing (MLST) to study genotypic relatedness among clinical *C. glabrata* isolates in Kuwait.

**Methods:**

*Candida glabrata* isolates (*n* = 91) cultured from 91 patients were analyzed by MLST. Repeat isolates (*n* = 16) from 9 patients were also used. Antifungal susceptibility testing for fluconazole, voriconazole, caspofungin and amphotericin B (AMB) was determined by Etest. Genetic relatedness was determined by constructing phylogenetic tree and minimum spanning tree by using BioNumerics software.

**Results:**

Resistance to fluconazole, voriconazole and AMB was detected in 7, 2 and 10 *C. glabrata* isolates, respectively. MLST identified 28 sequence types (STs), including 12 new STs. ST46 (*n* = 33), ST3 (*n* = 8), ST7 (*n* = 6) and ST55 (*n* = 6) were prevalent in ≥4 hospitals. Repeat isolates obtained from same or different site yielded identical ST. No association of ST46 with source of isolation or resistance to antifungals was apparent. Microevolution and cross-transmission of infection was indicated in two hospitals that yielded majority (57 of 91, 67%) of *C. glabrata*.

**Conclusion:**

Our data suggest that *C. glabrata* undergoes microevolution in hospital environment and can be nosocomially transmitted to other susceptible patients. Thus, proper infection control practices during routine procedures on *C. glabrata*-infected patients may prevent transmission of this pathogen to other hospitalized patients.

## 1. Introduction

Invasive fungal infections (IFIs) are mostly caused by opportunistic yeast pathogens in nosocomial settings and are associated with high mortality rates in critically ill patients (1–4). The incidence of IFIs is increasing globally due to increasing use of antifungal prophylaxis in an expanding population of immunocompromised/immunosuppressed individuals and other susceptible patients with multiple comorbidities, particularly in intensive care unit (ICU) settings (1, 2, 5, 6). Several novel yeasts with reduced susceptibility or resistance to antifungal drugs have emerged in recent years (7, 8). The increasing incidence of drug resistance in commonly encountered yeasts is also causing major shifts in the epidemiology of IFIs (5, 9–14). *Candida* spp. are also recognized as an important cause of late onset septicemia in low/very low-birth-weight neonates (15). Although *Candida albicans* is the most pathogenic and most frequently isolated species from clinical specimens, infections caused by non-albicans *Candida* species (NACS) have increased considerably in the last two decades and more than 50% of all *Candida* infections are now caused by NACS (5, 13, 16, 17). Widespread use of azole antifungal drugs and improvements in diagnostic methods have likely contributed to the increased proportion of NACS infections in recent years (5, 13, 18). *Candida glabrata*, along with two other closely related species; *Candida bracarensis* and *Candida nivariensis*, now form part of the *Nakaseomyces* clade, and hence, have recently been transferred to a new genus, *Nakaseomyces* (19). The emergence of *C. glabrata* (*N. glabrata*) as a common yeast pathogen is due to the intrinsic and acquired resistance of this species to azoles, the most widely used class of antifungal agents (20). *Candida glabrata* is the second or third most frequently isolated yeast species from blood and other clinical specimens from immunocompromised, cancer and diabetic patients, its isolation frequency is higher from older (>60 years) patients and those with indwelling medical devices and invasive infections are associated with higher mortality rates compared to infections with other *Candida* spp. (5, 13, 21, 22).

Opportunistic *Candida* infections in critically ill patients may be either endogenous or nosocomial in origin and this distinction is critical for effective intervention strategies (23–25). Several molecular methods have been used previously to determine genotypic similarities/differences among clinical *C. glabrata* strains (26–30). However, only few robust and/or portable methods namely, multi-locus sequence typing (MLST) based on sequence analyses of 5–7 conserved housekeeping loci, multi-locus microsatellite typing (MLMT), polymorphic locus sequence typing (PLST) and whole-genome sequencing (WGS) have been used in recent years (24, 25, 31). Although WGS provides the most comprehensive information and resolution, it is still costly for routine use in clinical settings and requires sophisticated data analyses while non-coding region-based methods (PLST) do not fulfill the evolutionary criteria which are exhibited by the conserved housekeeping loci (24, 26, 27). In this study, we performed MLST of *C. glabrata* isolates collected from hospitalized patients to determine its genetic structure, microevolution and nosocomial transmission of infection in Kuwait.

## 2. Materials and methods

### 2.1. Reference strains, clinical isolates, growth conditions and phenotypic and molecular identification

The *C. glabrata* (ATCC 90030 and CBS 135), *C. nivariensis* (CBS 9983), *C. bracarensis* (CBS 10154), *C. albicans* (ATCC 76615) and *Candida parapsilosis* (ATCC 22019) were used as reference strains. A total of 91 clinical *C. glabrata* isolates cultured from various clinical specimens and collected during 2007 to 2015 from 91 patients hospitalized in 9 different major government hospitals in Kuwait were used. These isolates were recovered from urine (*n* = 29), oral/respiratory specimens (*n* = 27 including 14 sputum, 9 tracheal aspirate, 3 oral swabs and 1 bronchoalveolar lavage samples), blood (*n* = 21), wound swab (*n* = 5), rectal swab (*n* = 4), pleural fluid/abdominal fluid/gastric aspirate (*n* = 3), axilla swab (*n* = 1) and vaginal swab (*n* = 1). Repeat isolates (*n* = 16) collected from the same or different anatomic sites of 9 patients were also used to ascertain the robustness of genotyping data.

The clinical specimens yielding *C. glabrata* were collected from the hospitalized patients after obtaining informed verbal consent only as part of routine diagnostic work-up for the isolation of bacterial and fungal pathogens. The data on deidentified samples are reported in this study. The clinical specimens were cultured by using BACTEC 9240 (Becton Dickinson, Sparks, MD, United States), BacT/Alert 3D (bioMérieux, Marcy-l’Étoile, France) and/or Versa TREK^™^240 (Thermo Fisher Scientific, United States) for the isolation of yeasts, as described previously (14, 17). All growth-positive cultures were subcultured on Sabouraud dextrose agar and Mast ID-CHROMagar Candida (Mast Diagnostics, Merseyside, United Kingdom) for phenotypic colony characteristics, as described previously (32). Species-specific identification was achieved by assimilation profiles obtained by commercial Vitek2 yeast identification system and/or by protein profiles by MALDI-TOF MS (bioMérieux, Marcy-l’Étoile, France), as described previously (33, 34). For molecular identification, the genomic DNA was extracted by using the Gentra Puregene Yeast DNA extraction kit (Qiagen, Hilden, Germany) according to kit instructions or by the rapid method using Chelex-100 as described previously (35). Molecular identification was performed by a multiplex PCR assay that discriminates *C. glabrata sensu stricto* from *C. nivariensis* and *C. bracarensis* among *C. glabrata sensu lato* strains, as described previously (36). The identity of 70 randomly selected isolates was further confirmed by DNA sequencing of internal transcribed spacer (ITS) region of rDNA by using panfungal ITS1 and ITS4 primers, performed as described previously (37). The study was approved by the Health Sciences Center Ethics Committee (vide approval No. VDR/EC/3724), Kuwait University and all the methods and investigations reported in this study were carried out according to their guidelines.

### 2.2. Antifungal susceptibility testing

*In vitro* antifungal susceptibility testing (AST) was conducted by Etest (bioMérieux SA, Marcy-l’Étoile, France) in accordance with the manufacturer’s instructions and as previously described (38). *C. parapsilosis* (ATCC22019) and *C. albicans* (ATCC90028) reference strains were employed for quality control. The minimum inhibitory concentration (MIC) values determined at the point of 80% growth inhibition for azoles and complete inhibition for amphotericin B (AMB) were read after 24 h. Interpretive susceptibility criteria for fluconazole (FLU), voriconazole (VOR) and caspofungin (CFG) were those recommended by Clinical and Laboratory Standards Institute (CLSI) (FLU: ≤32 μg/mL, susceptible dose-dependent, ≥64 μg/mL, resistant, VOR: ≤2 μg/mL, susceptible, >2 μg/mL, resistant, CFG: ≤0.12 μg/mL, susceptible, 0.25 μg/mL to <0.5 μg/mL, intermediate, ≥0.5 μg/mL, resistant) (39, 40) and for AMB were: ≤1 μg/mL, susceptible, >1 μg/mL, resistant (11).

### 2.3. Multi-locus sequence typing of *Candida glabrata* isolates

A total of six (*FKS*2, *LEU2*, *NMT1*, *TRP1*, *UGP1*, and *URA3*) housekeeping gene fragments accepted as an international standard for *C. glabrata* MLST^1^ were used (26). PCR was used to amplify each gene fragment from each isolate and PCR amplicons were purified by using PCR product purification kit (Qiagen Inc., Valencia, CA, United States) according to kit instructions. Both strands were sequenced using the BigDye terminator DNA sequencing kit (Applied Biosystems) with the same primers used for PCR amplification. The sequencing products were processed and loaded onto the DNA sequencer’s capillary as instructed by the manufacturer (Applied Biosystems) and as described previously (41). Clustal omega^2^ was used to verify forward and reverse allele profiles. The reproducibility of the MLST protocol was ensured by including the reference *C. glabrata* strain (ATCC 90030) in each run. The *C. glabrata* database was used to determine the allelic profile (allele number) for each gene. The allelic combinations for all the six loci for each isolate (sequence type, ST) were assigned or new allele and new STs were provided by the curator, Prof. Oliver Bader of the *C. glabrata* MLST database. The new allelic profiles and STs obtained in this study were submitted to the MLST database. The isolates belonged to the same ST if they contained the same allelic combinations for all the six loci. Based on the allele number for each isolate’s six gene fragments, a dendrogram was created using BioNumerics v7.5 (Applied Maths, Sint-Martens-Latem, Belgium) using the standard unweighted pair group method with arithmetic mean (UPGMA) parameters as described previously (41). A cluster was defined as a group of two or more *C. glabrata* isolates belonging to the same ST. The genetic relationship among the isolates was determined by constructing a minimum spanning tree using BioNumerics software which predicts putative relationships among the isolates and records two isolates as more closely related when five of six loci are identical (34, 41). Genotypic relationship between Kuwaiti and Asian *C. glabrata* isolates was also investigated.

### 2.4. Statistical analyses

The distribution of STs with respect to different clinical specimens or hospitals was compared by using Fisher’s exact test and probability levels <0.05 by the two-tailed test were considered as significant. Statistical analyses were performed by using WinPepi software ver. 11.65 (PEPI for Windows, Microsoft Inc., Redmond, WA, United States).

## 3. Results

### 3.1. Molecular identification of *Candida glabrata* isolates

A total of 1,149 *C. glabrata* isolates were cultured from 1,149 hospitalized patients in different government hospitals across Kuwait during 2007–2015. Repeat isolates cultured from the same and/or different anatomical sites were also available from some patients. More than 400 of these isolates were characterized in detail by various molecular methods including PCR-sequencing of rDNA (36). A total of 91 isolates from 91 patients and 16 repeat isolates from 9 of the same 91 patients were selected from this pool for fingerprinting studies by MLST (Supplementary Table S1). The largest number of isolates came from Hospital A (*n* = 33) followed by Hospital B (*n* = 24), Hospital C (*n* = 12), Hospital D (*n* = 7), Hospital E (*n* = 6), Hospital F (*n* = 4) and Hospital G (*n* = 3) while only one isolate each was included from Hospital H and Hospital I (Supplementary Table S1). The vast majority (74 of 91, 81.3%) of the isolates analyzed in this study were obtained in 2008 (*n* = 9), 2009 (*n* = 14), 2010 (*n* = 36) and 2011 (*n* = 15) and represented 20.4% (74 of 362) of all the isolates collected during this period. Repeat isolates (*n* = 9) from five (P26, P55, P56, P58, and P60) patients were from the same anatomical site as the first isolate while repeat isolates (*n* = 7) from four (P57, P59, P79 and P80) patients included isolates from different anatomical sites (Supplementary Table S1). All isolates were identified as *C. glabrata sensu lato* by Vitek2 yeast identification system and as *C. glabrata sensu stricto* by the MALDI-TOF MS and/or multiplex PCR. The identification was further confirmed by PCR-sequencing of 70 selected isolates as their ITS region of rDNA sequences showed <1% difference with the corresponding sequence from reference *C. glabrata* strains CBS135 and ATCC 90030, as expected.

### 3.2. Antifungal susceptibility testing of *Candida glabrata* isolates

The results of AST for *C. glabrata* isolates to four antifungal agents are shown in Table 1. The isolates exhibited MIC_50_ and MIC_90_ values of 0.11 and 1.5 μg/mL for AMB, 8 and 32 μg/mL for fluconazole, and 0.25 and 0.75 μg/mL for voriconazole, and 0.125 and 0.19 μg/mL for caspofungin, respectively. Majority (84 of 91, 92.3%) of *C. glabrata* isolates were susceptible dose-dependent while 7 isolates were resistant to fluconazole. Resistance to voriconazole was rare as only 2 isolates were resistant while the remaining 89 isolates were susceptible (Table 1). Eighty-one (89%) *C. glabrata* isolates were susceptible to AMB while 10 isolates were resistant. Eighty-three isolates were susceptible while 8 isolates were intermediate for caspofungin. Collectively, two (Kw1018/12 and Kw129/12) isolates were resistant to three drugs (AMB, fluconazole and voriconazole).

**Table 1:** Minimum inhibitory concentration (MIC) ranges and susceptibility data for 91 *Candida glabrata* isolates by Etest.

Antifungal agent	MIC_50_ μg/mL	MIC_90_ μg/mL	Range μg/mL	No. of isolates detected as		
				Susceptible	SDD/intermediate	Resistant
Amphotericin B	0.11	1.5	0.004–32	81	0	10
Fluconazole	8	32	0.016–256	0	84	7
Voriconazole	0.25	0.75	0.004–32	89	0	2
Capsofungin	0.125	0.19	0.006–0.5	83	8	0

Susceptibility breakpoints: amphotericin B: susceptible, ≤1 μg/mL, resistant >1 μg/mL, fluconazole: susceptible dose-dependent (SDD), ≤32 μg/mL, resistant ≥64 μg/mL, voriconazole: susceptible, ≤2 μg/mL, resistant >2 μg/mL, caspofungin: suscpetible, ≤0.25 μg/mL, intermediate, 0.25–0.5 μg/mL, resistant, ≥0.5 μg/mL.

### 3.3. Genotyping of *Candida glabrata* isolates by MLST

All six housekeeping gene fragments were successfully amplified by using the corresponding gene-specific forward and reverse primers. The DNA sequences were used to obtain the allelic profiles for each gene fragment and allele combinations to obtain STs from the *C. glabrata* MLST database. *TRP1* had the best typing efficiency (14 distinct genotypes/alleles), whereas *URA3* yielded the lowest typing efficiency (8 distinct genotypes/alleles). The polymorphic alleles combined to form 28 STs among 91 *C. glabrata* isolates from 91 patients yielding a ratio of number of STs to the number of *C. glabrata* isolates of 3.25 (one ST for every 3.25 isolates). The most prevalent ST was ST46 (33/91, 36.3%), followed by ST3 (8/91, 8.8%) and ST7 and ST55, each with a frequency of 6 (6.6%). Of the 28 STs, only 17 STs from 79 isolates were present in the *C. glabrata* MLST database while 11 STs (ST155 to ST165) from 12 isolates were new and were added to the *C. glabrata* MLST database. Sixteen (57.1%) of STs were represented by a single isolate which also represented 17.6% of the total 91 isolates. The allelic profile and final ST for each isolate are provided in Table 2. Repeat isolates of each patient collected from the same or different sites yielded the same allelic profile and ST as the first isolate. These included 5 bloodstream isolates from one (P60) patient and blood and non-blood isolates from three (P57, P79 and P80) patients. While the difference in the distribution of ST46, ST7 and ST55 among urine, oral/respiratory and blood specimens was not significant, the distribution of ST3 was significantly different in blood (4 of 21) compared to oral/respiratory specimens (0 of 27) (*p* = 0.031).

**Table 2:** Summary of fingerprinting data for 91 *C. glabrata* isolates using MLST.

ST	No. of isolates	*FKS2*	*LEU2*	*NMT1*	*TRP1*	*UGP1*	*URA3*
46	33	20	13	22	9	3	2
3	8	5	7	8	7	3	6
7	6	3	4	4	3	3	4
55	6	3	6	22	2	3	9
15	5	8	5	3	5	1	1
122	4	21	9	14	10	5	9
10	3	8	4	3	5	1	2
8	2	1	2	2	1	2	1
22	2	7	5	6	12	1	8
26	2	7	4	3	4	1	8
154	2	7	13	17	5	3	19
**162**	2	3	6	5	9	3	4
47	1	21	9	14	18	6	1
104	1	51	7	50	50	50	9
145	1	8	5	3	5	1	2
147	1	51	24	49	30	51	6
152	1	5	7	8	5	3	6
153	1	3	6	22	5	3	9
**155**	1	28	9	5	8	14	9
**156**	1	7	26	6	12	18	8
**157**	1	3	4	1	3	3	23
**158**	1	8	4	3	8	1	2
**159**	1	51	7	50	9	50	9
**160**	1	3	6	5	9	1	2
**161**	1	7	16	17	3	13	2
**163**	1	5	1	8	7	3	6
**164**	1	3	6	22	5	3	19
**165**	1	7	16	17	13	13	2

Bold and underlined numbers, new sequence types (STs) based on new alleles or new allelic combinations were found in this study.

The distribution of various STs among *C. glabrata* isolates collected from patients hospitalized in 9 hospitals are shown in Supplementary Table S1. The most common genotype, ST46 was found in eight hospitals while ST3, ST7, ST55, ST14 and ST122 were found in six, four, four, three and three hospitals, respectively (Supplementary Table S1). The distribution of four major genotypes (ST46, ST3, ST7 and ST55) among *C. glabrata* cultured at Hospital A, Hospital B and Hospital C which yielded the majority of the isolates was nearly same. The frequency of genotypic heterogeneity among *C. glabrata* isolates was nearly same (48.5% versus 54.2%) among isolates from the two major hospitals which contributed 63% of the total 91 isolates as 16 STs were detected among 33 isolates from Hospital A while 13 STs were found among 24 isolates from Hospital B (Supplementary Table S1). The frequency of genotypic heterogeneity was slightly higher among isolates from Hospital C (8 STs among 12 isolates, 66.7%), Hospital D (5 STs among 7 isolates, 71.4%) and Hospital E (4 STs among 6 isolates, 66.7%) (Supplementary Table S1). No specific association was found between a specimen type and ST as the six most common and previously reported STs (ST46, ST3, ST7, ST55, ST15 and ST122) were found among urine, bloodstream and oral/respiratory isolates. Similarly, there was no specific association of an ST with resistance of the isolate to an antifungal drug.

**Figure 1:**
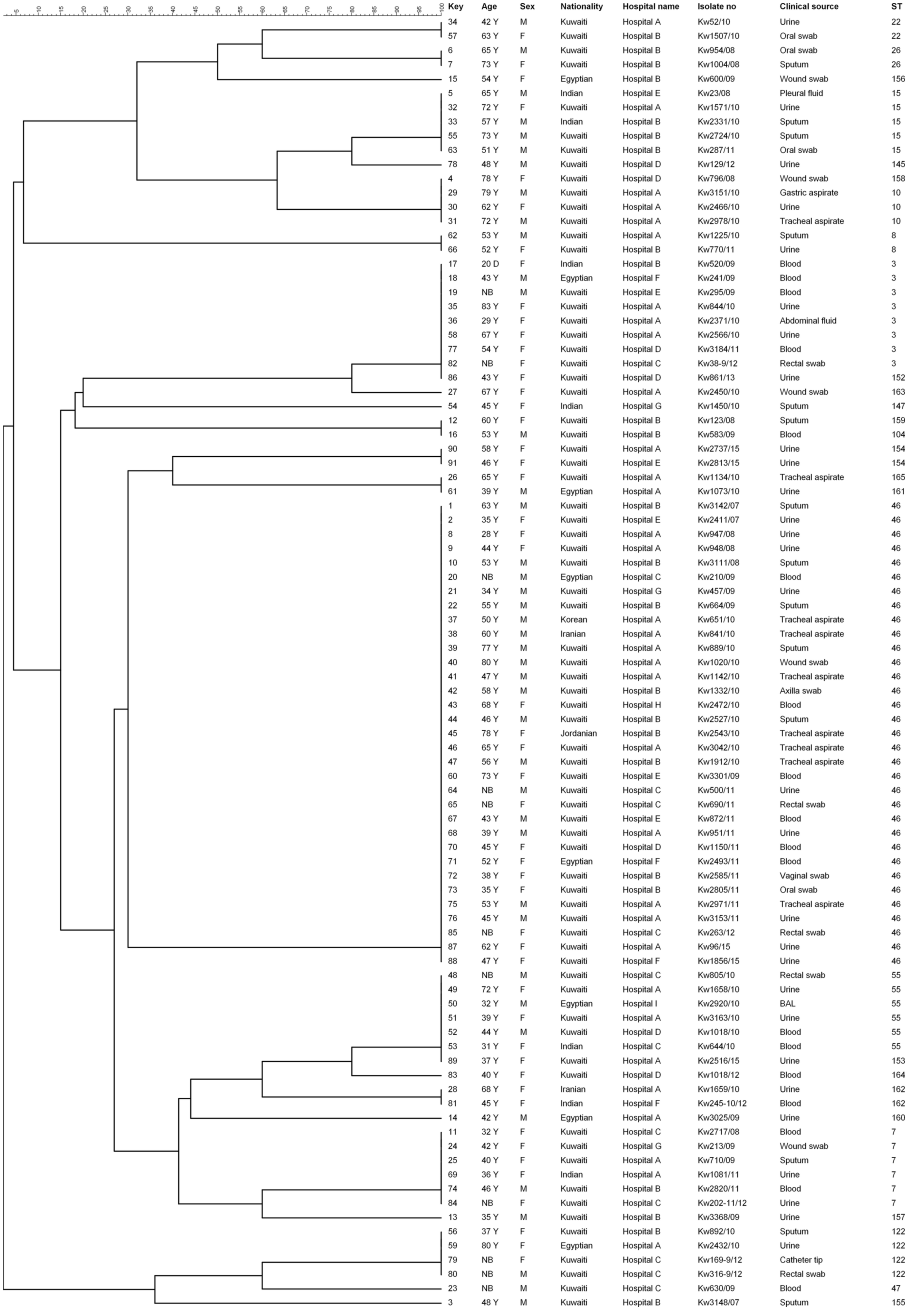
UPGMA tree based on MLST sequence data from 91 *Candida glabrata* isolates. The columns on left show MLST based sequence type (ST). Similarity is presented in percentages using the scale bar in the upper left corner. The columns from left to right include, patient number, age, sex, nationality, hospital name, isolate number, clinical source and MLST-based sequence type (ST).

The genetic association between the STs of *C. glabrata* isolates from Kuwait was determined by construction of an unrooted phylogenetic tree based on MLST data. The dendrogram (Figure 1) showed that 16 of 91 (17.6%) isolates were dispersed as unrelated singletons belonging to a single unique ST while the remaining 75 isolates typed into 13 clusters with the largest cluster (ST46) containing 33 of 75 (44%) of all cluster isolates. No obvious relationship was found between specific STs with patient’s nationality.

**Figure 2:**
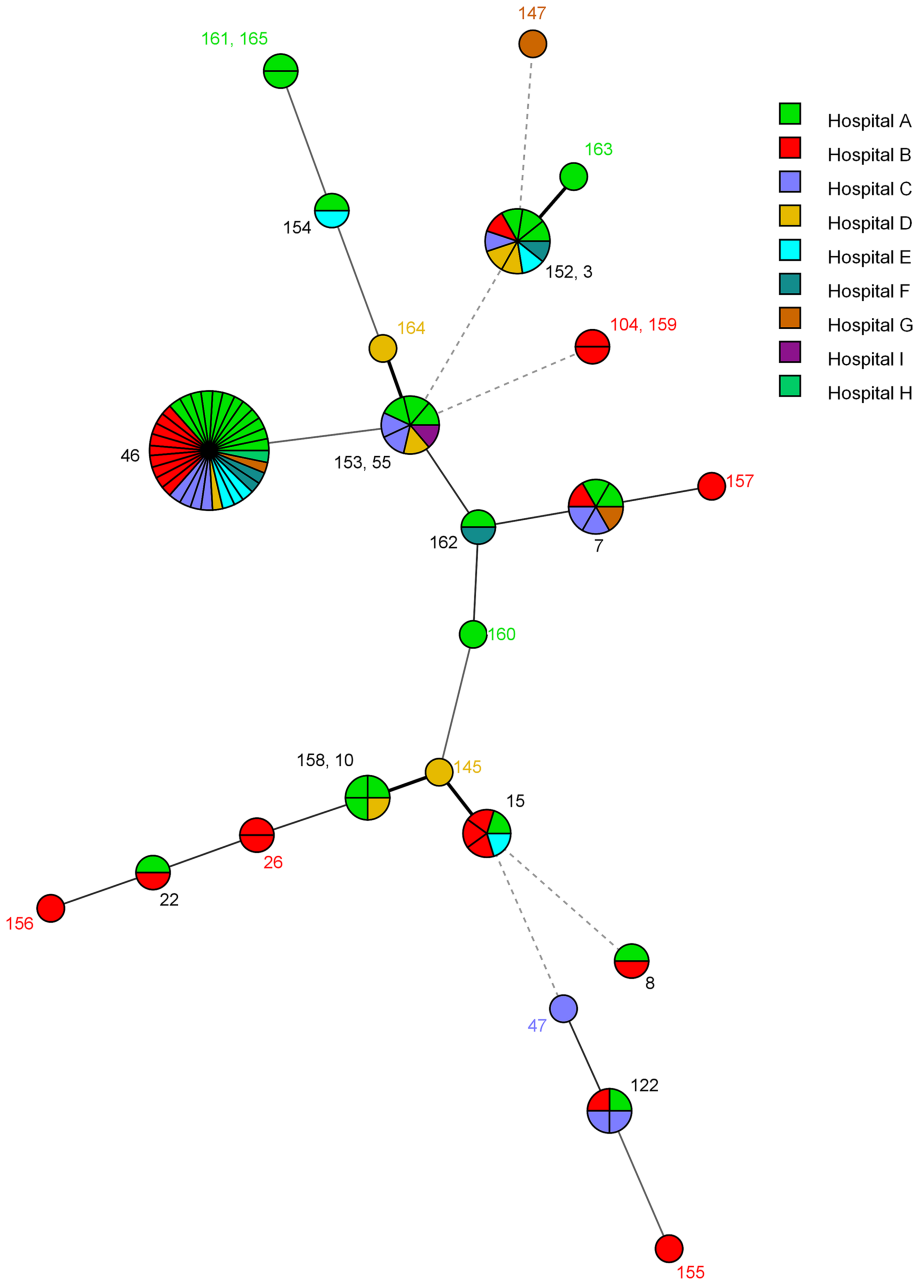
Minimum spanning tree showing genotypic relationship among *C. glabrata* isolated in different hospitals in Kuwait. Each circle corresponds to a unique genotype, and lines between circles represent relative distance between isolates. The sizes of the circles correspond to the number of isolates of the same genotype (ST). Connecting lines correspond to the number of allele differences between genotypes, with a solid thick line connecting genotypes that differ in one locus, a solid thin line connecting genotypes that differ in two-three loci, a dashed line connecting genotypes that differ in four loci, and a dotted line connecting genotypes that differ in more than four loci.

The microevolution and nosocomial transmission of *C. glabrata* isolates among hospitalized patients in Kuwait was also investigated by using the minimal spanning tree (MST) algorithm of the BioNumerics (v.7.6.1 software) software and the MST is depicted in Figure 2. The microevolution and nosocomial transmission of infection was more evident among *C. glabrata* isolates collected during 2008 to 2011 as majority (74 of 91, 81.3%) of the tested isolates were from this period and 57 of 91 (62.6%) isolates were cultured at Hospital A and Hospital B which are also located close (within 500 meters) to each other. Four specific cases of microevolution and nosocomial transmission of *C. glabrata* infection were recognized at Hospital A (Cases 1 to 3) and Hospital B (Case 4) which occurred within the same time-frame (±1 year) and are summarized in Table 3.

**Table 3:** Microevolution and cross transmission of *C. glabrata* among hospitalized patients in two major hospitals in Kuwait.

Case No.	Location	Year of isolation	Clinical specimen	Isolate No.	Multi-locus sequence typing-based allelic number for						Sequence type (ST)	Sequence of microevolution
					*FKS2*	*LEU2*	*NMT1*	*TRP1*	*UGP1*	*URA3*		
Case 1	Hospital A	2010	Urine	Kw844/10	5	7	8	7	3	6	ST3	ST3 to ST163
		2010	Abdominal fluid	Kw2371/10	5	7	8	7	3	6	ST3	
		2010	Urine	Kw2826/10	5	7	8	7	3	6	ST3^a^	
		2010	Wound swab	Kw2450/10	5	1	8	7	3	6	ST163^a^	
Case 2	Hospital A	2010	Urine	Kw1073/10	7	16	17	3	13	2	ST161^b^	ST161 to ST165
		2010	Tracheal aspirate	Kw1134/10	7	16	17	13	13	2	ST165^b^	
Case 3	Hospital A	2010	Urine	Kw1659/10	3	6	5	9	3	4	ST162^c^	ST162 to ST160
		2009	Urine	Kw3025/09	3	6	5	9	1	2	ST160^c^	
Case 4	Hospital B	2008	Sputum	Kw123/08	3	4	4	3	3	4	ST7^d^	ST7 to ST157
		2009	Blood	Kw583/09	3	4	1	3	3	27	ST157^d^	

^a,b,c,d^The ITS region of rDNA sequences were identical for the pair of isolates involved in microevolution in each of the four cases.

**Figure 3:**
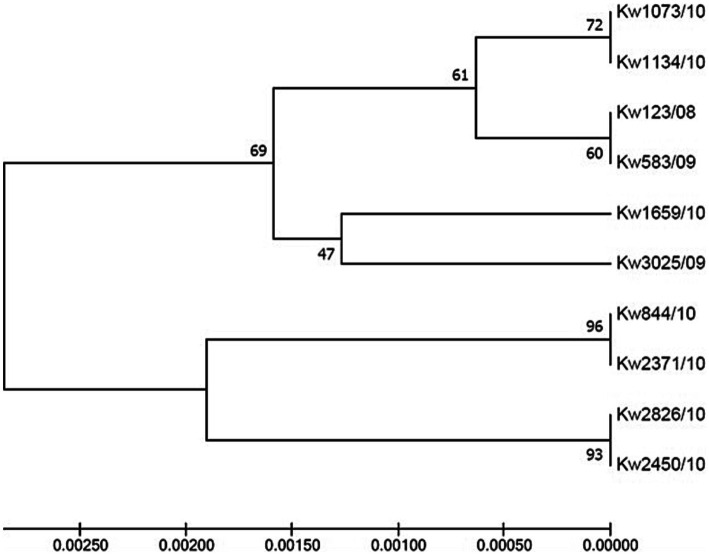
UPGMA tree based on internal transcribed spacer (ITS) region of rDNA sequence data from *C. glabrata* isolates. ITS sequences from four cases of *C. glabrata* (*n* = 10 isolates) with epidemiological linkage indicating microevolution and nosocomial transmission in the two hospitals in Kuwait.

Case 1 in Hospital A involved microevolution and nosocomial transmission of a *C. glabrata* isolate (Kw2450/10) obtained from wound and belonging to ST163 which evolved from an ST3 isolate obtained from urine (Kw2826/10) (Table 3). Case 2 involved a *C. glabrata* isolate (Kw1134/10) obtained from tracheal aspirate and belonging to ST165 which evolved from an ST161 urine isolate (Kw1073/10) while Case 3 involved two urine isolates, an ST160 isolate (Kw3025/09) which evolved from an ST162 isolate (Kw1659/10) (Table 3). Case 4 in Hospital B involved an ST157 *C. glabrata* urine isolate (Kw3368/09) which evolved from a bloodstream ST7 isolate (Kw2820/11) (Table 3). Interestingly, in all four cases, the two isolates contained identical sequence of the polymorphic ITS region of rDNA (Figure 3).

**Figure 4:**
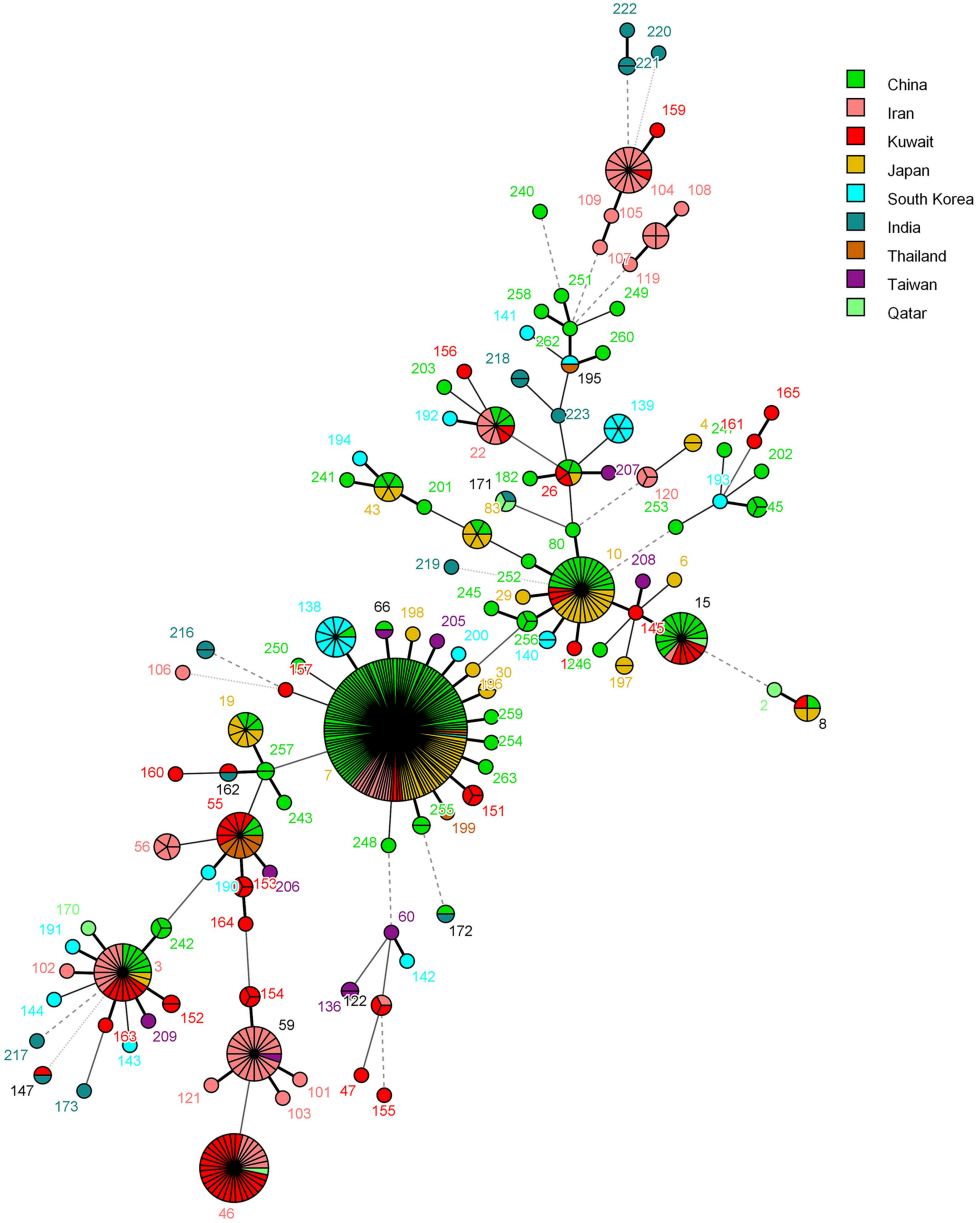
Minimum spanning tree showing relationship of *C. glabrata* from Kuwait with 498 isolates from other Asian countries available from the MLST website as of June 19, 2023. Each circle corresponds to a unique genotype, and lines between circles represent relative distance between isolates. The sizes of the circles correspond to the number of isolates of the same genotype (ST). Connecting lines correspond to the number of allele differences between genotypes, with a solid thick line connecting genotypes that differ in one locus, a solid thin line connecting genotypes that differ in two-three loci, a dashed line connecting genotypes that differ in four loci, and a dotted line connecting genotypes that differ in more than four loci.

The population structure of *C. glabrata* isolates from Kuwait was also investigated by determining their genetic relationship with isolates from other Asian countries in the central data library of the *C. glabrata* MLST database by using the MST and the data are depicted in Figure 4. Most of the isolates from Kuwait clustered with isolates from Iran while few isolates also clustered with *C. glabrata* isolates from India, Japan, and China (Figure 4).

## 4. Discussion

The IFIs are increasing worldwide and are mostly caused by opportunistic *Candida* and other yeast species in nosocomial settings (1, 2, 5–8). Opportunistic *Candida* infections may be either endogenous or nosocomial in origin and this distinction is critical for effective intervention strategies. Molecular fingerprinting studies are crucial in determining the source of infection and nosocomial transmission of yeast pathogens in healthcare settings (25, 31, 34, 41, 42). Fingerprinting studies of *Candida* spp. isolates have mostly been carried out by employing MLST or MLMT-based methods (28–31, 34, 41–43). Although MLST schemes were devised for *C. albicans* (44) and *C. glabrata* (26) at nearly the same time, extensive studies have been carried out on clinical *C. albicans* isolates while only few studies have explored the population structure of *C. glabrata* (28, 31, 45–53).

*C. glabrata* is among the most frequently isolated NACS from clinical specimens in Kuwait (17, 18). A total of 91 *C. glabrata* isolates collected during 2007 to 2015 were selected for MLST analyses. Although these represented only ~8% of the total 1,149 isolates collected during 2007–2015, significantly higher number of isolates were analyzed from the years 2008 to 2011 as 74 of 362 (20.4%) isolates were included for MLST studies. MLST identified 28 STs among 91 isolates giving a ratio (number of STs to the number of isolates) of 3.25 which is nearly same as that reported from Australia (2.88, 18 STs among 52 isolates) (50), Iran (3.12, 16 STs among 50 isolates) (51) and Tanzania (3.6, 13 STs among 47 isolates) (52). On the contrary, a higher ratio of 5.3 (15 STs among 80 isolates) has been reported from Taiwan (46), 7.41 (31 STs among 230 isolates) (47) and 9.13 (22 STs among 201 isolates) (48) among *C. glabrata* isolates from the United States, 6.97 (30 STs among 209 isolates) from Republic of Korea (49) and 7.82 (17 STs among 133 isolates) (28) and 11.4 (35 STs among 411 isolates) (53) from China. The higher genetic diversity of *C. glabrata* from Kuwait, an Arabian Gulf country much smaller than Australia, Iran or Tanzania, is likely due to the large (nearly three times of Kuwaiti nationals) and highly diverse expatriate population (54, 55). This is also supported by the relatively higher frequency (16 of 91, 17.6%) of *C. glabrata* isolates from Kuwait represented by a single and unique ST. Similar values have also been reported from Australia (8 of 52, 15.3%) (50) and Iran (9 of 50, 18%) (51) while lower frequencies have been reported from Taiwan (6 of 80, 7.5%) (46), Republic of Korea (20 of 209, 9.6%) (49), China (23 of 411, 5.6%) (28) and (7 of 133, 5.2%) (53) and from the United States (16 of 230, 6.9%) (47) and (7 of 201, 3.5%) (48).

Although high genetic diversity was evident among 91 *C. glabrata* isolates collected over an extended period-of-time (2007 to 2015) from Kuwait, ST46 was the dominant genotype detected in 33 of 91 (36.3%) isolates and this genotype was present in nearly all hospitals and all major specimen types. Interestingly, ST46 has been detected previously in other countries, however, it was not found as the dominant genotype previously, including studies from other (Iran and Qatar) Middle Eastern countries (31, 51). On the contrary, ST7 is the predominant genotype in East Asian countries as this genotype was detected in 23 of 80 (28.7%) *C. glabrata* isolates from Taiwan (46), 100 of 209 (47.8%) isolates from Republic of Korea (49), and 273 of 411 (66.4%) (28) and 81 of 133 (60.9%) (53) isolates from China. The second most common genotype from Kuwait, ST3, has been reported as the most common genotype among *C. glabrata* isolates from Australia (8 of 51, 15.7%) (50) and United States (38 of 201, 18.1%) (48), it is the second most common genotype among isolates from Taiwan (13 of 80, 16.2%) (46), Republic of Korea (47 of 209, 22.5%) (49) and China (39 of 411, 9.5%) (28) and has also been detected among the isolates from Iran (4 of 50, 8%) (51). The ST3 is also a common genotype among *C. glabrata* isolates from Belgium, France, Germany, Spain and the United Kingdom (26, 50), reflecting the universal spreading of this genotype. Other common genotypes (ST55, ST15 and ST10) detected in this study also appear to have a worldwide distribution as they have been detected previously in several other countries, including, Australia, China, France, Japan, Iran, Qatar, Spain, Taiwan, Tanzania, United Kingdom and the United States (26, 28, 31, 47–53).

The distribution of three major genotypes (ST46, ST7 and ST55) among urine, oral/respiratory and blood specimens was not significantly different, however, ST3 was not detected among oral/respiratory specimens. There was no association of a particular ST with resistance of *C. glabrata* isolates in Kuwait to an antifungal drug. Only few previous studies have shown an association of specific STs with resistance of *C. glabrata* to antifungal drugs. Fluconazole resistant *C. glabrata* isolates from China were associated with ST7 and/or ST3 (28, 53) while ST18 isolates from Tanzania exhibited lower mean fluconazole susceptibility than isolates belonging to other STs (52).

Repeat *C. glabrata* isolates from the same patient obtained from the same site in Kuwait yielded the same ST. Only few studies have performed MLST on repeat isolates from the same patient. The only repeat isolate in the study from Australia belonged to the same genotype (ST8) as the first isolate (50). Lin et al. (46), analyzed multiple isolates from 25 patients and their data showed that repeat isolates from 19 patients belonged to the same ST or very closely related genotype. However, multiple isolates from 6 patients belonged to distinct STs (46). Our study also showed that bloodstream *C. glabrata* isolates from three patients belonged to the same ST as non-blood isolates obtained from the catheter tip (patient P79) or mucosal sites (oral cavity and rectum) (patients P57 and P80, respectively). These findings suggest translocation of *C. glabrata* from mucosal sites to bloodstream causing candidemia in these three patients. Similar findings have also been recorded recently among patients colonized with multidrug-resistant *Candida auris* who later developed candidemia (56, 57). A total of 5 isolates were analyzed from a patient (P60) with persistent bloodstream infection and all isolates belonged to ST46. A recent study showed that multiple *C. glabrata* colonies from candidemia patients exhibited heterogenous susceptibility results but were indistinguishable by MLST as they all belonged to ST3 (58). Unfortunately, whole genome sequencing (WGS) was not performed on *C. glabrata* isolates described by Knoll et al. (58) as well as on the ST46 isolates from P60 in our study. However, a more recent study employing WGS has shown that *C. glabrata* isolates from candidemia patients contain mixed populations of clonal but genetically diverse strains which cannot be differentiated by MLST alone (59). These findings suggest that MLST likely records events which happened several months/years ago while WGS can detect changes occurring in the same patient over days/weeks.

An interesting observation of our study is the detection of microevolution and nosocomial transmission of *C. glabrata* in the two major hospitals which yielded most of the isolates analyzed in this study. Three specific cases of microevolution and nosocomial transmission of *C. glabrata* were detected in Hospital A and one in Hospital B. In each case, the two isolates contained identical sequence of the ITS region of rDNA which, unlike other *Candida* spp., is often variable among clinical *C. glabrata sensu stricto* isolates (36, 43, 60). Although clinical details of patients, disease severity and outcome were not available, there was no apparent relationship among microevolution-related *C. glabrata* isolates with respect to the age or gender of patients. Nosocomial transmission of *C. glabrata*, initially indicated by PLST (27), was recently demonstrated for cluster P and cluster N isolates in a recent study by including additional analyses of another highly polymorphic (CgMT-C) locus (25). Although not specifically stated, microevolution of *C. glabrata* isolate belonging to ST182 from an isolate of ST26 and its nosocomial transmission was also evident among the two strains (L255 and L256) isolated from the two patients in LH Hospital in a recent study from China (53).

Our study has a few limitations. (1) Only 91 of the total 1,149 (7.9%) *C. glabrata* isolates collected during 2007 to 2015 in Kuwait were analyzed by MLST. These isolates were selected from >400 of *C. glabrata* isolates that were recently characterized in detail by various molecular methods including PCR-sequencing of rDNA. (2) The distribution of the isolates for some years and from some hospitals was too small to draw any meaningful conclusions for these settings. (3) The microevolution and nosocomial transmission of *C. glabrata* suggested by MLST analyses was not confirmed by WGS studies. (4) The clinical details of patients, disease severity and outcome were not available. Since MLST is labor intensive and time consuming, we intend to extend these observations by performing WGS on *C. glabrata* isolates collected more recently.

## 5. Conclusion

We have performed molecular fingerprinting of *C. glabrata* isolates by multilocus sequence typing for the first time in Kuwait and the Arabian Peninsula. Our results have identified a dominant genotype (ST46) among *C. glabrata* isolates in Kuwait and have also suggested that *C. glabrata* undergoes microevolution in the hospital environment and is nosocomially transmitted to other susceptible patients. Further investigations by WGS are needed to confirm these findings. If WGS confirms microevolution and nosocomial transmission of infection, proper infection control practices during routine procedures on *C. glabrata*-infected patients will greatly help in preventing transmission of this pathogen to other hospitalized patients.

## Data availability statement

All relevant data described in the paper are present within the manuscript and in the supplementary material. The rDNA sequence data have been submitted to GenBank under accession no. FN652301, LS398116, LT837722, LT837724 and OR226607-OR226612 and are freely available. The sequence data for different alleles comprising new MLST STs reported in this study have also been submitted to the *C. glabrata* MLST database (https://pubmlst.org/bigsdb?db=pubmlst_cglabrata_seqdef) which are also freely available.

## Ethics statement

The studies involving humans were approved by Ethics Committee of the Health Sciences Center, Kuwait University. The studies were conducted in accordance with the local legislation and institutional requirements. The human samples used in this study were acquired as a by-product of routine patient care. Written informed consent for participation was not required from the participants or the participants’ legal guardians/next of kin in accordance with the national legislation and institutional requirements.

## Author contributions

MA, SA, NA-S, and ZK conceptualized the research and wrote the manuscript. MA, SA, and ZK supervised the study, designed the experiments, and interpreted the data. All authors contributed to the article and approved the submitted version.

## Funding

The study was supported by the Department of Microbiology, College of Medicine, Kuwait University and Research Core Facility grant SRUL02/13.

## Conflict of interest

The authors declare that the research was conducted in the absence of any commercial or financial relationships that could be construed as a potential conflict of interest.

## Publisher’s note

All claims expressed in this article are solely those of the authors and do not necessarily represent those of their affiliated organizations, or those of the publisher, the editors and the reviewers. Any product that may be evaluated in this article, or claim that may be made by its manufacturer, is not guaranteed or endorsed by the publisher.

## Footnotes

^1^ https://pubmlst.org/organisms/candida-glabrata

^2^ http://www.ebi.ac.uk/Tools/msa/clustalo/
