# Supplementary material for: Molecular fingerprinting by multi-locus sequence typing identifies microevolution and nosocomial transmission of Candida glabrata in Kuwait
Source: Front Public Health. 2023 Sep 8;11:1242622. doi: 10.3389/fpubh.2023.1242622 (PMC10515652; doi:10.3389/fpubh.2023.1242622)
Supplement: Supplementary file 1 [file Table_1.docx]

S1 Table. Demographic and other details of patients and fingerprinting data for 91 *C. glabrata* and 16 duplicate isolates obtained from 91 patients from Kuwait analyzed by MLST

| **Patient no.** | **Age^a^** | **Sex** | **Nationality** | **Hospital name** | **Isolate no.** | **Clinical source^b^** | **FKS2** | **LEU2** | **NMT1** | **TRP1** | **UGP1** | **URA3** | **ST^c^** |
| --- | --- | --- | --- | --- | --- | --- | --- | --- | --- | --- | --- | --- | --- |
| 1 | 63 Y | M | Kuwaiti | Hospital B | Kw3142/07 | Sputum | 20 | 13 | 22 | 9 | 3 | 2 | 46 |
| 2 | 35 Y | F | Kuwaiti | Hospital E | Kw2411/07 | Urine | 20 | 13 | 22 | 9 | 3 | 2 | 46 |
| 3 | 48 Y | M | Kuwaiti | Hospital B | Kw3148/07 | Sputum | 28 | 9 | 5 | 8 | 14 | 9 | **155** |
| 4 | 78 Y | F | Kuwaiti | Hospital D | Kw796/08 | Wound swab | 8 | 4 | 3 | 8 | 1 | 2 | **158** |
| 5 | 65 Y | M | Indian | Hospital E | Kw23/08 | Pleural fluid | 8 | 5 | 3 | 5 | 1 | 1 | 15 |
| 6 | 65 Y | M | Kuwaiti | Hospital B | Kw954/08 | Oral swab | 7 | 4 | 3 | 4 | 1 | 8 | 26 |
| 7 | 73 Y | F | Kuwaiti | Hospital B | Kw1004/08 | Sputum | 7 | 4 | 3 | 4 | 1 | 8 | 26 |
| 8 | 28 Y | F | Kuwaiti | Hospital A | Kw947/08 | Urine | 20 | 13 | 22 | 9 | 3 | 2 | 46 |
| 9 | 44 Y | F | Kuwaiti | Hospital A | Kw948/08 | Urine | 20 | 13 | 22 | 9 | 3 | 2 | 46 |
| 10 | 53 Y | M | Kuwaiti | Hospital B | Kw3111/08 | Sputum | 20 | 13 | 22 | 9 | 3 | 2 | 46 |
| 11 | 32 Y | F | Kuwaiti | Hospital C | Kw2717/08 | Blood | 3 | 4 | 4 | 3 | 3 | 4 | 7 |
| 12 | 60 Y | F | Kuwaiti | Hospital B | Kw123/08 | Sputum | 51 | 7 | 50 | 9 | 50 | 9 | **159** |
| 13 | 35 Y | M | Kuwaiti | Hospital B | Kw3368/09 | Urine | 3 | 4 | 1 | 3 | 3 | 23 | **157** |
| 14 | 42 Y | M | Egyptian | Hospital A | Kw3025/09 | Urine | 3 | 6 | 5 | 9 | 1 | 2 | **160** |
| 15 | 54 Y | F | Egyptian | Hospital B | Kw600/09 | Wound swab | 7 | 26 | 6 | 12 | 18 | 8 | **156** |
| 16 | 53 Y | M | Kuwaiti | Hospital B | Kw583/09 | Blood | 51 | 7 | 50 | 50 | 50 | 9 | 104 |
| 17 | 20 D | F | Indian | Hospital B | Kw520/09 | Blood | 5 | 7 | 8 | 7 | 3 | 6 | 3 |
| 18 | 43 Y | M | Egyptian | Hospital F | Kw241/09 | Blood | 5 | 7 | 8 | 7 | 3 | 6 | 3 |
| 19 | NB | M | Kuwaiti | Hospital E | Kw295/09 | Blood | 5 | 7 | 8 | 7 | 3 | 6 | 3 |
| 20 | NB | M | Egyptian | Hospital C | Kw210/09 | Blood | 20 | 13 | 22 | 9 | 3 | 2 | 46 |
| 21 | 34 Y | M | Kuwaiti | Hospital G | Kw457/09 | Urine | 20 | 13 | 22 | 9 | 3 | 2 | 46 |
| 22 | 55 Y | M | Kuwaiti | Hospital B | Kw664/09 | Sputum | 20 | 13 | 22 | 9 | 3 | 2 | 46 |
| 23 | NB | M | Kuwaiti | Hospital C | Kw630/09 | Blood | 21 | 9 | 14 | 18 | 6 | 1 | 47 |
| 24 | 42 Y | F | Kuwaiti | Hospital G | Kw213/09 | Wound swab | 3 | 4 | 4 | 3 | 3 | 4 | 7 |
| 25 | 40 Y | F | Kuwaiti | Hospital A | Kw710/09 | Sputum | 3 | 4 | 4 | 3 | 3 | 4 | 7 |
| 26 | 65 Y | F | Kuwaiti | Hospital A | Kw1134/10 | TA | 7 | 16 | 17 | 13 | 13 | 2 | **165** |
| 26 dup | 65 Y | F | Kuwaiti | Hospital A | Kw1182/10 | Throat swab | 7 | 16 | 17 | 13 | 13 | 2 | **165** |
| 27 | 67 Y | F | Kuwaiti | Hospital A | Kw2450/10 | Wound swab | 5 | 1 | 8 | 7 | 3 | 6 | **163** |
| 28 | 68 Y | F | Iranian | Hospital A | Kw1659/10 | Urine | 3 | 6 | 5 | 9 | 3 | 4 | **162** |
| 29 | 79 Y | M | Kuwaiti | Hospital A | Kw3151/10 | Gastric aspirate | 8 | 4 | 3 | 5 | 1 | 2 | 10 |
| 30 | 62 Y | F | Kuwaiti | Hospital A | Kw2466/10 | Urine | 8 | 4 | 3 | 5 | 1 | 2 | 10 |
| 31 | 72 Y | M | Kuwaiti | Hospital A | Kw2978/10 | TA | 8 | 4 | 3 | 5 | 1 | 2 | 10 |
| 32 | 72 Y | F | Kuwaiti | Hospital A | Kw1571/10 | Urine | 8 | 5 | 3 | 5 | 1 | 1 | 15 |
| 33 | 57 Y | M | Indian | Hospital B | Kw2331/10 | Sputum | 8 | 5 | 3 | 5 | 1 | 1 | 15 |
| 34 | 42 Y | M | Kuwaiti | Hospital A | Kw52/10 | Urine | 7 | 5 | 6 | 12 | 1 | 8 | 22 |
| 35 | 83 Y | F | Kuwaiti | Hospital A | Kw844/10 | Urine | 5 | 7 | 8 | 7 | 3 | 6 | 3 |
| 36 | 29 Y | F | Kuwaiti | Hospital A | Kw2371/10 | Abdominal fluid | 5 | 7 | 8 | 7 | 3 | 6 | 3 |
| 37 | 50 Y | M | Korean | Hospital A | Kw651/10 | TA | 20 | 13 | 22 | 9 | 3 | 2 | 46 |
| 38 | 60 Y | M | Iranian | Hospital A | Kw841/10 | TA | 20 | 13 | 22 | 9 | 3 | 2 | 46 |
| 39 | 77 Y | M | Kuwaiti | Hospital A | Kw889/10 | Sputum | 20 | 13 | 22 | 9 | 3 | 2 | 46 |
| 40 | 80 Y | M | Kuwaiti | Hospital A | Kw1020/10 | Wound swab | 20 | 13 | 22 | 9 | 3 | 2 | 46 |
| 41 | 47 Y | M | Kuwaiti | Hospital A | Kw1142/10 | TA | 20 | 13 | 22 | 9 | 3 | 2 | 46 |
| 42 | 58 Y | M | Kuwaiti | Hospital B | Kw1332/10 | Axilla swab | 20 | 13 | 22 | 9 | 3 | 2 | 46 |
| 43 | 68 Y | F | Kuwaiti | Hospital H | Kw2472/10 | Blood | 20 | 13 | 22 | 9 | 3 | 2 | 46 |
| 44 | 46 Y | M | Kuwaiti | Hospital B | Kw2527/10 | Sputum | 20 | 13 | 22 | 9 | 3 | 2 | 46 |
| 45 | 78 Y | F | Jordanian | Hospital B | Kw2543/10 | TA | 20 | 13 | 22 | 9 | 3 | 2 | 46 |
| 46 | 65 Y | F | Kuwaiti | Hospital A | Kw3042/10 | TA | 20 | 13 | 22 | 9 | 3 | 2 | 46 |
| 47 | 56 Y | M | Kuwaiti | Hospital B | Kw1912/10 | TA | 20 | 13 | 22 | 9 | 3 | 2 | 46 |
| 48 | NB | M | Kuwaiti | Hospital C | Kw805/10 | Rectal swab | 3 | 6 | 22 | 2 | 3 | 9 | 55 |
| 49 | 72 Y | F | Kuwaiti | Hospital A | Kw1658/10 | Urine | 3 | 6 | 22 | 2 | 3 | 9 | 55 |
| 50 | 32 Y | M | Egyptian | Hospital I | Kw2920/10 | BAL | 3 | 6 | 22 | 2 | 3 | 9 | 55 |
| 51 | 39 Y | F | Kuwaiti | Hospital A | Kw3163/10 | Urine | 3 | 6 | 22 | 2 | 3 | 9 | 55 |
| 52 | 44 Y | M | Kuwaiti | Hospital D | Kw1018/10 | Blood | 3 | 6 | 22 | 2 | 3 | 9 | 55 |
| 53 | 31 Y | F | Indian | Hospital C | Kw644/10 | Blood | 3 | 6 | 22 | 2 | 3 | 9 | 55 |
| 54 | 45 Y | F | Indian | Hospital G | Kw1450/10 | Sputum | 51 | 24 | 49 | 30 | 51 | 6 | 147 |
| 55 | 73 Y | M | Kuwaiti | Hospital B | Kw2724/10 | Sputum | 8 | 5 | 3 | 5 | 1 | 1 | 15 |
| 55 dup | 73 Y | M | Kuwaiti | Hospital B | Kw2793/10 | Oral swab | 8 | 5 | 3 | 5 | 1 | 1 | 15 |
| 56 | 37 Y | F | Kuwaiti | Hospital B | Kw892/10 | Sputum | 21 | 9 | 14 | 10 | 5 | 9 | 122 |
| 56 dup1 | 37 Y | F | Kuwaiti | Hospital B | Kw1040/10 | Sputum | 21 | 9 | 14 | 10 | 5 | 9 | 122 |
| 56 dup2 | 37 Y | F | Kuwaiti | Hospital B | Kw1051/10 | Sputum | 21 | 9 | 14 | 10 | 5 | 9 | 122 |
| 57 | 63 Y | F | Kuwaiti | Hospital B | Kw1507/10 | Oral swab | 7 | 5 | 6 | 12 | 1 | 8 | 22 |
| 57 dup1 | 63 Y | F | Kuwaiti | Hospital B | Kw1617/10 | Blood | 7 | 5 | 6 | 12 | 1 | 8 | 22 |
| 57 dup2 | 63 Y | F | Kuwaiti | Hospital B | Kw1640/10 | ET aspirate | 7 | 5 | 6 | 12 | 1 | 8 | 22 |
| 58 | 67 Y | F | Kuwaiti | Hospital A | Kw2566/10 | Urine | 5 | 7 | 8 | 7 | 3 | 6 | 3 |
| 58 dup | 67 Y | F | Kuwaiti | Hospital A | Kw2826/10 | Urine | 5 | 7 | 8 | 7 | 3 | 6 | 3 |
| 59 | 80 Y | F | Egyptian | Hospital A | Kw2432/10 | Urine | 21 | 9 | 14 | 10 | 5 | 9 | 122 |
| 59 dup1 | 80 Y | F | Egyptian | Hospital A | Kw2863/10 | Urine | 21 | 9 | 14 | 10 | 5 | 9 | 122 |
| 59 dup2 | 80 Y | F | Egyptian | Hospital A | Kw3046/10 | Wound swab | 21 | 9 | 14 | 10 | 5 | 9 | 122 |
| 60 | 73 Y | F | Kuwaiti | Hospital E | Kw3301/09 | Blood | 20 | 13 | 22 | 9 | 3 | 2 | 46 |
| 60 dup1 | 73 Y | F | Kuwaiti | Hospital E | Kw3376/09 | Blood | 20 | 13 | 22 | 9 | 3 | 2 | 46 |
| 60 dup2 | 73 Y | F | Kuwaiti | Hospital E | Kw3411/09 | Blood | 20 | 13 | 22 | 9 | 3 | 2 | 46 |
| 60 dup3 | 73 Y | F | Kuwaiti | Hospital E | Kw790/10 | Blood | 20 | 13 | 22 | 9 | 3 | 2 | 46 |
| 60 dup4 | 73 Y | F | Kuwaiti | Hospital E | Kw929/10 | Blood | 20 | 13 | 22 | 9 | 3 | 2 | 46 |
| 61 | 39 Y | M | Egyptian | Hospital A | Kw1073/10 | Urine | 7 | 16 | 17 | 3 | 13 | 2 | **161** |
| 62 | 53 Y | M | Kuwaiti | Hospital A | Kw1225/10 | Sputum | 1 | 2 | 2 | 1 | 2 | 1 | 8 |
| 63 | 51 Y | M | Kuwaiti | Hospital B | Kw287/11 | Oral swab | 8 | 5 | 3 | 5 | 1 | 1 | 15 |
| 64 | NB | M | Kuwaiti | Hospital C | Kw500/11 | Urine | 20 | 13 | 22 | 9 | 3 | 2 | 46 |
| 65 | NB | F | Kuwaiti | Hospital C | Kw690/11 | Rectal swab | 20 | 13 | 22 | 9 | 3 | 2 | 46 |
| 66 | 52 Y | F | Kuwaiti | Hospital B | Kw770/11 | Urine | 1 | 2 | 2 | 1 | 2 | 1 | 8 |
| 67 | 43 Y | M | Kuwaiti | Hospital E | Kw872/11 | Blood | 20 | 13 | 22 | 9 | 3 | 2 | 46 |
| 68 | 39 Y | M | Kuwaiti | Hospital A | Kw951/11 | Urine | 20 | 13 | 22 | 9 | 3 | 2 | 46 |
| 69 | 36 Y | F | Indian | Hospital A | Kw1081/11 | Urine | 3 | 4 | 4 | 3 | 3 | 4 | 7 |
| 70 | 45 Y | F | Kuwaiti | Hospital D | Kw1150/11 | Blood | 20 | 13 | 22 | 9 | 3 | 2 | 46 |
| 71 | 52 Y | F | Egyptian | Hospital F | Kw2493/11 | Blood | 20 | 13 | 22 | 9 | 3 | 2 | 46 |
| 72 | 38 Y | F | Kuwaiti | Hospital B | Kw2585/11 | Vaginal swab | 20 | 13 | 22 | 9 | 3 | 2 | 46 |
| 73 | 35 Y | F | Kuwaiti | Hospital B | Kw2805/11 | Oral swab | 20 | 13 | 22 | 9 | 3 | 2 | 46 |
| 74 | 46 Y | M | Kuwaiti | Hospital B | Kw2820/11 | Blood | 3 | 4 | 4 | 3 | 3 | 4 | 7 |
| 75 | 53 Y | M | Kuwaiti | Hospital A | Kw2971/11 | TA | 20 | 13 | 22 | 9 | 3 | 2 | 46 |
| 76 | 45 Y | M | Kuwaiti | Hospital A | Kw3153/11 | Urine | 20 | 13 | 22 | 9 | 3 | 2 | 46 |
| 77 | 54 Y | F | Kuwaiti | Hospital D | Kw3184/11 | Blood | 5 | 7 | 8 | 7 | 3 | 6 | 3 |
| 78 | 48 Y | M | Kuwaiti | Hospital D | Kw129/12 | Urine | 8 | 5 | 3 | 5 | 1 | 2 | 145 |
| 79 | NB | F | Kuwaiti | Hospital C | Kw169-9/12 | Catheter tip | 21 | 9 | 14 | 10 | 5 | 9 | 122 |
| 79 dup | NB | F | Kuwaiti | Hospital C | Kw170-9/12 | Blood | 21 | 9 | 14 | 10 | 5 | 9 | 122 |
| 80 | NB | M | Kuwaiti | Hospital C | Kw316-9/12 | Rectal swab | 21 | 9 | 14 | 10 | 5 | 9 | 122 |
| 80 dup1 | NB | M | Kuwaiti | Hospital C | Kw317-9/12 | Blood | 21 | 9 | 14 | 10 | 5 | 9 | 122 |
| 80 dup2 | NB | M | Kuwaiti | Hospital C | Kw73-10/12 | Blood | 21 | 9 | 14 | 10 | 5 | 9 | 122 |
| 81 | 45 Y | F | Indian | Hospital F | Kw245-10/12 | Blood | 3 | 6 | 5 | 9 | 3 | 4 | **162** |
| 82 | NB | F | Kuwaiti | Hospital C | Kw38-9/12 | Rectal swab | 5 | 7 | 8 | 7 | 3 | 6 | 3 |
| 83 | 40 Y | F | Kuwaiti | Hospital D | Kw1018/12 | Blood | 3 | 6 | 22 | 5 | 3 | 19 | **164** |
| 84 | NB | F | Kuwaiti | Hospital C | Kw202-11/12 | Urine | 3 | 4 | 4 | 3 | 3 | 4 | 7 |
| 85 | NB | F | Kuwaiti | Hospital C | Kw263/12 | Rectal swab | 20 | 13 | 22 | 9 | 3 | 2 | 46 |
| 86 | 43 Y | F | Kuwaiti | Hospital D | Kw861/13 | Urine | 5 | 7 | 8 | 5 | 3 | 6 | 152 |
| 87 | 62 Y | F | Kuwaiti | Hospital A | Kw96/15 | Urine | 20 | 13 | 22 | 9 | 3 | 2 | 46 |
| 88 | 47 Y | F | Kuwaiti | Hospital F | Kw1856/15 | Urine | 20 | 13 | 22 | 9 | 3 | 2 | 46 |
| 89 | 37 Y | F | Kuwaiti | Hospital A | Kw2516/15 | Urine | 3 | 6 | 22 | 5 | 3 | 9 | 153 |
| 90 | 58 Y | F | Kuwaiti | Hospital A | Kw2737/15 | Urine | 7 | 13 | 17 | 5 | 3 | 19 | 154 |
| 91 | 46 Y | F | Kuwaiti | Hospital E | Kw2813/15 | Urine | 7 | 13 | 17 | 5 | 3 | 19 | 154 |

^a^ D, day, Y, year, NB, newborn, dup, duplicate isolate

^b^ TA, tracheal aspirate, BAL, bronchoalveolar lavage

^c^ Bold ST, new sequence type found in this study
